# Involvement of PI3K/Akt signaling pathway in promoting osteogenesis on titanium implant surfaces modified with novel non-thermal atmospheric plasma

**DOI:** 10.3389/fbioe.2022.975840

**Published:** 2022-09-16

**Authors:** Zheng Zheng, Yanjin He, Li Long, Shuaiqi Gan, Shujiang Chen, Min Zhang, Jia Xu, Ruijie Fu, Yihan Liao, Zhimin Zhu, Hang Wang, Wenchuan Chen

**Affiliations:** ^1^ State Key Laboratory of Oral Diseases, National Clinical Research Center for Oral Diseases, West China Hospital of Stomatology, Sichuan University, Chengdu, China; ^2^ Department of Oral Prosthodontics, West China Hospital of Stomatology, Sichuan University, Chengdu, Sichuan, China

**Keywords:** non-thermal plasma, titanium, implant, surface modification, PI3K/Akt signaling pathway, osteogenesis

## Abstract

Non-thermal atmospheric plasma (NTAP) modification to induce a hydrophilic titanium (Ti) surface with less carbon contamination, has been demonstrated to boost the osteogenic responses. In this study, we investigated the underlying bone formation mechanism of NTAP-Ti, and the involvement of PI3K/Akt signaling pathway in regulating osteogenic activities on NTAP-Ti surfaces. NTAP was employed for Ti activation, and PI3K inhibitor, LY294002, was applied to the suppression of PI3K/Akt pathway. We systematically and quantitatively detected the cell morphology, attachment, proliferation, osteogenic differentiation and mineralization of MC3T3-E1 mouse preosteoblasts, and molecular expressions involved in osteogenesis and PI3K/Akt signaling pathway *in vivo* and *in vitro*. A descent in osteoblast proliferation on Ti surfaces in relation to LY294002. Alkaline phosphatase (ALP) activity, as well as matrix mineralization, was mitigated by PI3K inhibitor in NTAP-Ti. Likewise, the expression levels of osteogenesis-related genes [ALP, osteocalcin (Ocn), osteopontin (Opn) and runt-related transcription factor 2 (Runx2)] on NTAP-Ti were notably attenuated by LY294002, as confirmed by the results of osteogenesis-related proteins (ALP, and Runx2) expression analysis. In addition, the expression of PI3K/Akt signal pathway proteins further verified the inhibition of LY294002 on Ti surfaces modified by NTAP. Collectively, the PI3K/Akt signal pathway was involved in the amelioration of osteogenesis induced by NTAP modification. NTAP treatment for Ti activation is promising in augmented osteogenic potential through the activation of PI3K/Akt signal pathway.

## Introduction

Titanium (Ti) implants has become a valid and well-documented treatment in dental and orthopedic clinical applications (Kaur and Singh, 2019). Implant restoration has become an indispensable part of modern medicine. The global dental implant market is rising rapidly and is projected to reach about $13.01 billion by the year 2023 (Alghamdi and Jansen, 2020). However, Ti surfaces incline to adsorb organic impurities from ambient atmosphere, as a result of function variations in Ti surface characteristics (Suzuki et al., 2010). This phenomenon represents that conventional implant surfaces’ functional drawbacks are involved with hydrophobicity and contaminations, which further diminish the biologically available clean surface areas, and result in unfavorable peri-implant biological behaviors. The physical, chemical and biological performances of material surfaces are closely related to biological interactions between implants and surrounding tissues, and the degree of bone-implant integration that is achieved (Civantos et al., 2017). Given that, various material surface modification techniques have been investigated to promote the peri-implant osteogenesis. Among them, surface modifications, leading to an increased hydrophilicity, have been demonstrated to further the early healing stages of peri-implant tissue, accelerate the speed of osseointegration and reduce treatment duration (Eriksson et al., 2004; Klein et al., 2013).

The hydroxylated/hydrated titanium surfaces were produced with hydrophilic character (Schwarz et al., 2009). A hydrophilic implant surface was characterized by a low carbon concentration and high levels of oxygen (Sartoretto et al., 2015). This hydrophilic surface directly impacted on initial protein-to-surface interactions that might manage the osteoblast adhesion and osteoblastic differentiation process, and regulate peri-implant tissue formation (Chappuis et al., 2018). It was proved that hydrophilic implants had successfully been applied to irradiated, diabetes mellitus and osteoporosis patients (Heberer et al., 2011; Mardas et al., 2011; Schlegel et al., 2013). This treatment also expanded clinical indications to a great extent, simultaneously also obviously reduced the incidence of complications and relieved patients’ discomfort. The hydrophilic implants are prepared by sandblasting and acid-etching under nitrogen protection, followed by storage in isotonic saline (Buser et al., 2004). In addition, these implants could be provided by chair-side treatment with aqueous sodium hydroxide (Tugulu et al., 2010). However, drawbacks such as high technical difficulty, unstable performance of hydrophilicity, and associated high costs have restricted their clinical applications.

Plasma treatment is a feasible solution to the adversities mentioned above. It is ionized gas generated by electric discharges, including mixtures of heavy (molecules, atoms, free radicals, ions) and light (electrons and photons) species. These accessible and efficient plasma devices feature relatively low manufacturing costs (Braný et al., 2020). Recently, non-thermal atmospheric plasma (NTAP) has been developed under atmospheric pressure at a low temperature, and applied in several industries, as well as in biology and medicine (Tanaka and Hori, 2017). NTAP functionalization was capable of endowing enhanced surface wettability and energy, thus leading to super-hydrophilicity, without adverse impact on surface topography (Henningsen et al., 2018). In the prior study, we developed novel apparatus and methods of NTAP treatment with a special clamping device using mixed gas (argon/oxygen) for Ti activation (Zheng et al., 2020). We have investigated the NTAP-Ti’s surface properties, and assessed the attractiveness for cells and early osseointegration on NTAP-Ti surfaces *in vitro* and *in vivo*. It was clear that hydrophilic NTAP-Ti surfaces facilitated the osteogenic properties and integration with bone. However, the exact molecular mechanisms of osteoblasts in response to Ti surfaces hydrophilicity activated by NTAP, still remain vague. Therefore, the elucidation of the correlation among NTAP, certain signal pathway and osteogenesis could provide progressive comprehension of the theories how Ti surface performances involved with osteogenic activities. An in-depth study into how NTAP promote the osteogenic procedures will further assist in improving the applications of hydrophilic implants.

Generally, the biological effect of osteogenesis correlates closely with certain specific cellular signaling pathways. Among them, the phosphatidylinositol-3-kinase (PI3K)/protein kinase B (Akt) signaling pathway performs critical functions in biological behavior of osteoblasts and bone formation, by regulating fundamental cellular processes (Schultze et al., 2012). In addition, surface hydrophilicity boosts bone formation by means of directly enhancing an earlier expression of pathways with respect to cell proliferation and osteoblast precursor differentiation (e.g., PI3K/Akt, Wnt, Notch, and transforming growth factor-β) (Calciolari et al., 2018). PI3K is a heterodimeric enzyme composed of a catalytic (p110) and a regulatory subunit (p85), important for a diverse spectrum of cellular activities, including metabolism and aging, proliferation and oncogenicity (Ito et al., 2018). Moreover, Akt is a downstream phosphoinositide-dependent serine-threonine protein kinase, activated through its binding to PI3K, imposing a conformational change and affording the subsequent phosphorylation of two different residues (Ser473, Thr308) within the molecule (Risso et al., 2015). It was certified that PI3K/Akt pathway and its downstream targets were crucial regulators of bone absorption by osteoclasts and bone formation by osteoblasts, through boosting their differentiation and survival to maintain bone mass and turnover (Mukherjee and Rotwein, 2012). Akt deficiency in osteoblasts resulted in increased susceptibility to apoptosis, and suppressed differentiation and function (Kawamura et al., 2007). Briefly, PI3K/Akt pathway plays a key role in the process of bone formation on hydrophilic surface.

Therefore, we postulated that the favorable osteogenic effect of NTAP-Ti may be mediated by activation of PI3K/Akt signaling pathway, which has been not distinctly demonstrated. On the basis of the preliminary study that the conducive osteogenic competence of hydrophilic NTAP-Ti’s surface has been proved, the purposes of the present study were to: 1) initially identify the underlying mechanism of NTAP-Ti on osteogenesis; 2) determine the interaction among NTAP-Ti, PI3K/Akt signaling pathway, and osteogenesis. The following hypothesis was tested: PI3K/Akt signaling pathway would be engaged in promoting osteogenesis on NTAP-Ti. It is prospective that our study would provide a helpful demonstration for the application of NTAP in implant restoration.

## Materials and methods

### NTAP source

The novel apparatus and methods using NTAP for Ti activation were developed and described earlier in the previous study (Zheng et al., 2020). The NTAP system incorporate the alternating current supply, collet, quartz tube, copper tube, and working gases. In this device, NTAP produced by dielectric barrier discharge under argon gas at a flow rate of 3,000 standard cubic centimeters per minute (sccm) and oxygen levels at 0.5%. The composition and flow rate of the working gases were precisely controlled by two mass flow controllers (Laifeng Technology Co., Ltd., Chengdu, China).

### Sample preparation

Commercial-grade pure Ti disks (c.p. Ti, grade IV, 99.9 mass%, Xinhangfeng Technology, Chengdu, China) were prepared in 15 mm diameter and 2 mm thick. Ti samples were cleaned ultrasonically with 75% ethanol for 30 min, then rinsed with DI water for 30 min, and ultimately disinfected in autoclaves for at least 1 h. All Ti samples were divided into four groups: 1) Ti control group (Ti), untreated by NTAP; 2) Ti control group with PI3K inhibitor (Ti + L), untreated by NTAP and provided with LY294002; 3) Ti experimental group (NTAP-Ti, N-Ti for short), processed by NTAP for 60 s; 4) Ti experimental group with PI3K inhibitor (NTAP-Ti + L, N-Ti + L for short), processed by NTAP for 60 s and provided with LY294002.

### Cell culture

MC3T3-E1 mouse preosteoblasts were supplied by the State Key Laboratory of Oral Diseases (Sichuan University, China). All cell experiments were authorized by the institutional review board of Sichuan University. Cells were incubated with alpha-minimum essential medium (α-MEM, Gibco, Gaithersburg, MD, United States) containing 10% Fetal bovine serum (FBS, EVERY GREEN, Zhejiao Tianhang biotechnology Co., Ltd., China) and 1% Penicillin/streptomycin (PS, HyClone, Logan, UT, United States). PI3K inhibitor, LY294002 (S1737, Beyotime Institute of Biotechnology, Shanghai, China) was added to the cell medium at concentration of 10 μM for 1 d (Wang et al., 2017). The inhibitor concentration was confirmed on the basis of manufacturer’s recommendation and earlier published studies. Cultures were conducted at 37°C in an incubator with a humidified atmosphere of 5% CO_2_. The MC3T3-E1 cells were sub-cultured using trypsin digestion, and then seeded at the density of 1.0 × 10^4^ cells/disk on the Ti samples in a 24-well polystyrene plate.

### Cell morphology

After incubation for 12 h, MC3T3-E1 cell morphology on Ti surfaces was visualized by field emission scanning electron microscopy (FE-SEM, FEI Inspect F, United States) with an accelerating voltage of 20.0 kV. At 24 h, TRITC Phalloidin and DAPI (Solarbio Bioscience & Technology Co., Ltd., Shanghai, China) were used to label the cellular actin filaments and cell nucleus, respectively. The stained MC3T3-E1 cells were observed in an inverted fluorescence microscope (Leica DMi8, Germany). The MC3T3-E1 cells’ area and density were measured using ImageJ software (version 1.51v, Bethesda, MD).

### Cell proliferation assay

Cell counting kit-8 (CCK-8) was purchased from Dojindo (Kyushu, Japan). CCK-8 assay was used to estimate MC3T3-E1 cell proliferation on Ti disks after 1 d, 3 d and 5 d of culture. At each time point, the cells were cleaned twice with PBS and added with fresh culture medium containing the CCK-8 solution (10% of cell culture medium). After incubation at 37°C for 2 h without light, the absorbance at 450 nm of the solution was measured using a Varioskan LUX Multimode Microplate Reader (Thermo Scientific, Madison, WI, United States) (Shao et al., 2021).

### Alkaline phosphatase activity assay

After 24 h of culture, the medium was replaced with an osteogenic medium supplemented with 10 mM β-glycerol phosphate, 0.2 mM ascorbic acid and 10–4 mM dexamethasone for the following osteogenic-related studies. The osteogenic medium was replaced every 2 d. After 4 d, 7 d and 14 d of culture, MC3T3-E1 cells were lysed with 1% Triton X-100. The lysates were centrifuged, and the supernatants were reacted with para-nitrophenyl phosphate (pNPP) substrate from an Alkaline Phosphatase Assay Kit (P0321, ALP Assay Kit, Beyotime Institute of Biotechnology, Shanghai, China) at 37°C in the dark for 30 min. The absorbance of para-nitrophenol (p-nitrophenol) released was measured at 405 nm on a microplate reader. The ALP activity was expressed as the amount of containing p-nitrophenol. The ALP activity was standardized by the total protein content measured using a bicinchoninic acid (BCA) protein assay kit (P0010, Beyotime Institute of Biotechnology, Shanghai, China) (St-Pierre et al., 2005).

### Evaluation for calcification

Alizarin red S (ARS) staining was applied to observe calcium nodules and measure extracellular calcium deposits generated by MC3T3-E1 cells. After 7 d, 14 d and 21 d of osteogenic differentiation, the cells were fixed with 4% PFA for 30 min and stained with ARS solution (1%, pH4.2, Solarbio, China) for 5 min at room temperature. The cells were washed three times with PBS and observed under a stereomicroscope (Olympus, Japan). The alizarin red S-stained cells were destained with a 10% cetylpyridium chloride and incubated for 60 min at 37°C. The absorbance at 562 nm of the solution was measured using a microplate reader (Li and Wang, 2020).

### Gene expression analysis

The expression of osteogenic-related genes in MC3T3-E1 cells was determined using quantitative reverse transcription polymerase chain reaction (qRT-PCR). Total RNA was extracted using a Trizol reagent (Thermo Scientific, Madison, WI, United States) after 4 d, 7 d and 14 d of osteogenic differentiation. The RNA concentrations were determined by NanoPhotometer-N50 (Implen, Germany) and adjusted to 0.25 μg/μL by diluting with DNase/RNase-free water (Tiangen Biotech Co., Ltd., Beijing, China). The total RNA was reverse-transcribed, and complementary DNA (cDNA) was synthesized using a RevertAid First Strand cDNA Synthesis Kit (Thermo Scientific, Madison, WI, United States) in a final volume of 20 μL. The mRNA expressions of ALP, Ocn, Opn and Runx2 were evaluated by qRT-PCR using 2 × T5 Fast qPCR Mix (SYBRGreenI, Tsingke Biological Technology Co., Ltd., Beijing, China). Reactions were performed in 20 μL with approximately 100 ng cDNA in QuantStudio three Real-Time PCR System (Applied Biosystems, Thermo Fisher Scientific, Inc.). qRT-PCR conditions were 5 s at 95°C for denaturation and 34 s at 60°C for annealing and extension, for a total of 40 cycles. The mRNA expressions of ALP, Ocn, Opn and Runx2 were normalized on that of the reference gene β-actin. The relative mRNA expression was analyzed and calculated using the 2^−ΔΔCt^ method (Chen et al., 2018; Su et al., 2022). The sequences of the primers used were listed in Table 1.

**TABLE 1:** The primers sequences used for qRT-PCR.

Gene	Forward primer sequence (5′-3′)	Reverse primer sequence (5′-3′)
ALP	CTG​CCT​GAA​ACA​GAA​AGT​CTG​C	TAT​GTC​TTT​ACC​AGG​AGG​CGT​G
Ocn	GGA​CCA​TCT​TTC​TGC​TCA​CTC​TG	ACC​TTA​TTG​CCC​TCC​TGC​TTG
Opn	TTC​TCC​TGG​CTG​AAT​TCT​GAG​G	GCT​GCC​AGA​ATC​AGT​CAC​TTT​C
Runx2	ACG​AAA​AAT​TAA​CGC​CAG​TCG​G	CAC​TTC​ACC​CTC​AGG​ACC​G
β-actin	AGA​TTA​CTG​CTC​TGG​CTC​CTA​GC	ACT​CAT​CGT​ACT​CCT​GCT​TGC​T

### Protein expression analysis

The protein expressions involved in osteogenesis and PI3K/Akt signaling pathway were detected by Western blot. After 7 d of incubation, MC3T3-E1 cells were washed with PBS twice, harvested and lysed in cell lysis buffer for Western (RIPA Lysis Buffer (Strong), APExBIO, Houston, TX, United States). All protein samples were separated using 10% SDS-polyacrylamide gel electrophoresis (SDS-PAGE) and transferred to polyvinylidene fluoride (PVDF) membranes (Millipore, MA, United States) that could adsorb the protein with non-covalently and retain the type of polypeptide and biological activity after separated by electrophoresis. After blocking, the PVDF membranes were respectively incubated with primary antibodies, including Anti-rabbit ALP, Runx2, PI3K, phospho-PI3K (p-PI3K), Akt, phospho-Akt (p-Akt), and Anti-mouse β-actin, overnight at 4°C. Anti-ALP (SA40-00) was purchased from HUABIO; Anti-Runx2 (12556S), PI3K (4292S), p-PI3K (4228S), Akt (4691S), and p-Akt (4060S) antibodies were from Cell Signaling Technology; Anti-β-actin (66009-1-Ig) was from ProteinTech. After being incubated with the corresponding secondary antibodies for 1 h, the immunoreactive protein components were detected using an enhanced chemiluminescence (ECL) kit (US Everbright Inc., China), and the quantification of bands was evaluated using the VersaDocTM imaging system (Bio-Rad, Hercules, CA, United States) (Li and Wang, 2020).

### Animals and surgery

The animal experiment was submitted to and approved by State Key Laboratory of Oral Diseases & National Clinical Research Center for Oral Diseases (WCHSIRB-D-2017-285), according to the ARRIVE (Animals in Research: Reporting *In Vivo* Experiments) guidelines. The bilateral maxillary first molars (M1) of four-week-old male Sprague–Dawley rats (*n* = 15, mean weight: 90–110 g) were extracted. After 4 weeks, the implants with or without NTAP treatment were placed into the extracted M1 sites. After surgery, prophylactic antibiotics (80,000 units per day, Penicillin Potassium, Solarbio, China) were performed daily for 3 days. At 6 weeks of healing, maxillae containing a cylindrical implant were harvested.

### Immunohistochemical analysis

All maxillae were fixed in 4% paraformaldehyde overnight, and then decalcified with 10% ethylenediaminetetraacetic acid for 30 days. The implants were removed before samples were embedded in paraffin. 5-μm sections were prepared for immunohistochemical staining of p-Akt. Stained sections were observed using a light microscope (Leica, Germany). The p-Akt-positive cells were calculated using ImageJ software (version 1.51v, Bethesda, United States).

### Statistical analyses

IBM SPSS 22.0 statistical software was applied to executing all statistical analyses. Data were presented as the mean ± standard deviation (SD). Statistical differences and multiple comparisons among different groups were measured by using one-way analysis of variance (ANOVA) and factorial analysis. The difference was deemed statistically significant when *p* value was less than 0.05.

## Results

### Influence on the morphology, adhesion and proliferation of MC3T3-E1 cells

**FIGURE 1:**
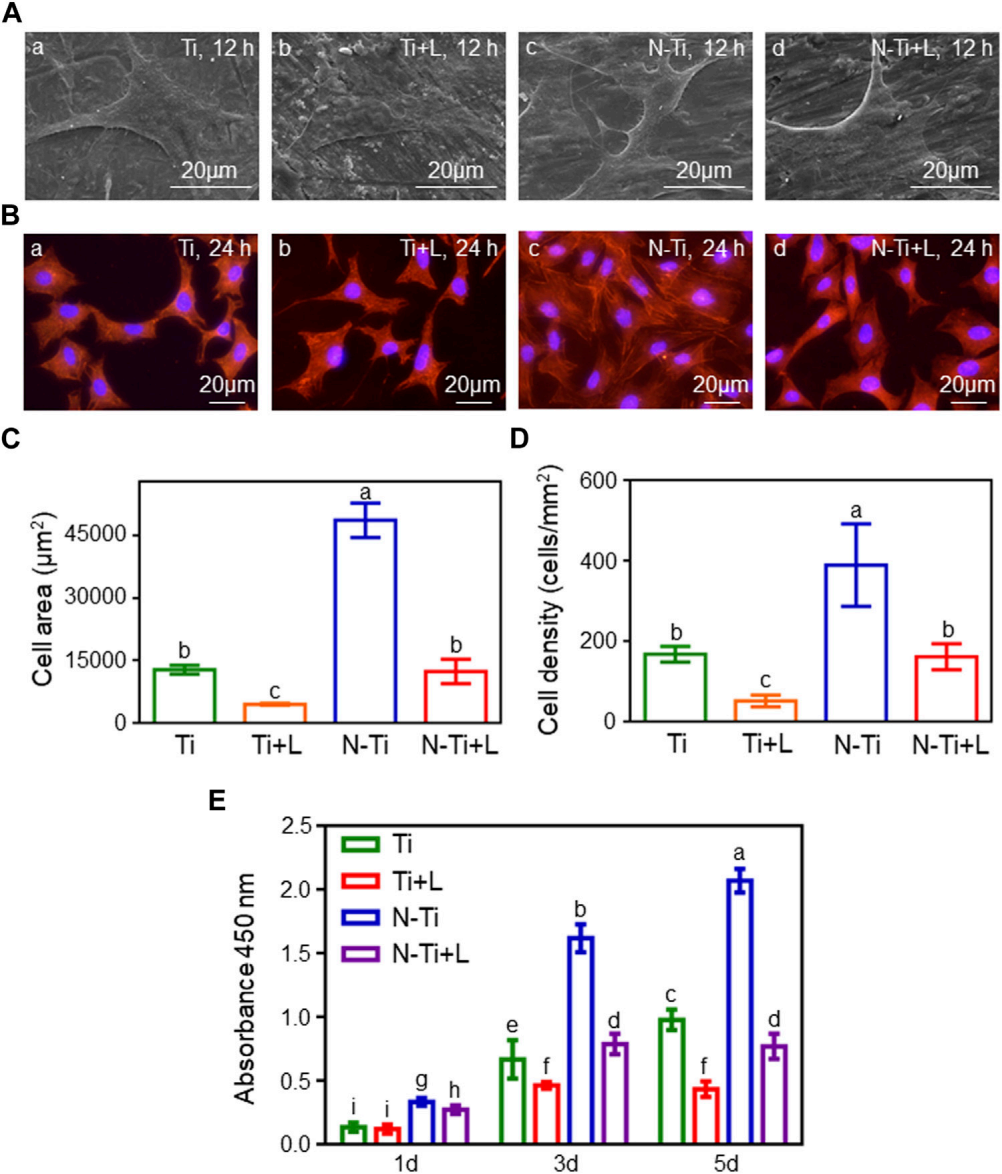
Cell morphology, adhesion and proliferation of MC3T3-E1 cells. **(A)** Cell morphology of MC3T3-E1 cells cultured on Ti, Ti + L, N-Ti, N-Ti + L surface after 12 h of incubation. **(B)** TRITC Phalloidin, and DAPI labeled MC3T3-E1 cells on Ti surfaces at 24 h. Area **(C)** and density **(D)** of MC3T3-E1 cells were analyzed quantitatively. **(E)** Cell proliferation of MC3T3-E1 cells on Ti samples surface after 1 d, 3 d and 5 d of culture. Results are shown as mean ± SD; *n* = 6/group. Values with diverse letters are significantly different (*p* < 0.05).

MC3T3-E1 cell morphology was observed using FE-SEM and a fluorescence microscope. After 12 and 24 h of culture, the numbers of adherent MC3T3-E1 cells notably declined with the addition of LY294002 (Figures 1A,B). The polygonal cells on NTAP-Ti surface displayed a spreading morphology with more protruded pseudopodia than other groups. At 24 h, the mean MC3T3-E1 cell area showed a 75 and 65% descent in N-Ti + L and Ti + L compared with N-Ti and Ti groups, respectively (*p* < 0.05) (Figure 1C). Meanwhile, the cell density was decreased by 60 and 70% in LY294002 groups (Figure 1D).

Cell proliferation was tested at 1, 3 and 5 d after inoculation by CCK-8 assay (Figure 1E). As the incubation time was increased, the absorbances at 450 nm of adhered MC3T3-E1 cells progressively gained with the highest levels of proliferation seen on the N-Ti surface, following by Ti and N-Ti + L, and then Ti + L. In the early stage of incubation at 1 d, the absorbances at 450 nm in N-Ti and Ti groups were augmented than N-Ti + L and Ti + L groups, respectively (*p* < 0.05). The N-Ti and Ti group leaded to a 105 and 45%, 170 and 120% enhancement of the absorbances at 450 nm, when compared to the N-Ti + L and Ti + L at 3 and 5 d, respectively (*p* < 0.05).

### Influence on the osteogenic differentiation of MC3T3-E1 cells

**FIGURE 2:**
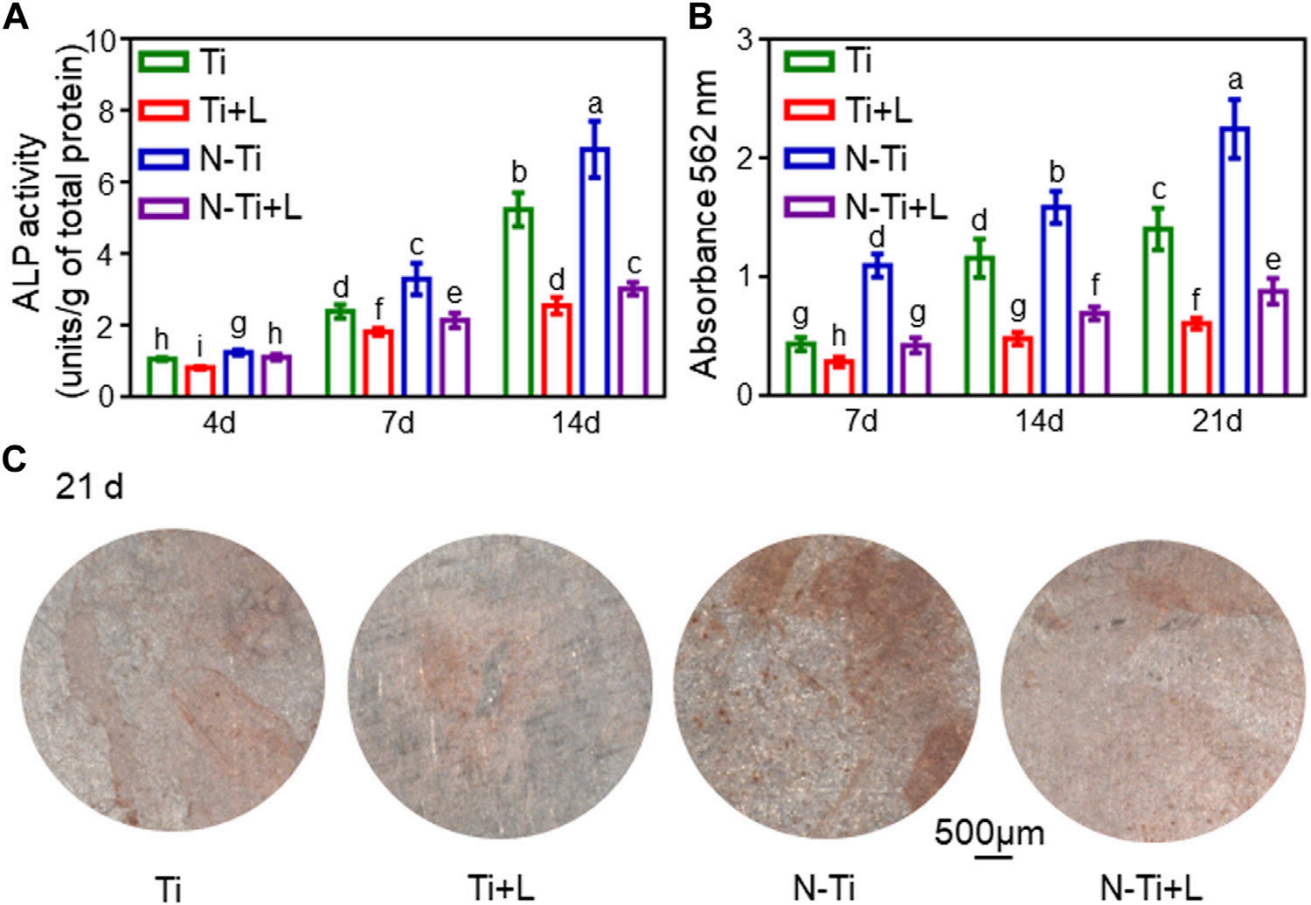
Osteogenic differentiation and mineralization of MC3T3-E1 cells on Ti, Ti + L, N-Ti, N-Ti + L surfaces. **(A)** ALP activity of MC3T3-E1 cells seeded on Ti surfaces for 4, 7, and 14 d. **(B)** Colorimetric quantitative results of mineralization. **(C)** ARS staining of MC3T3-E1 cells incubated on Ti surfaces for 21 d. Results are shown as mean ± SD; *n* = 6/group. Values with dissimilar letters are markedly different (*p* < 0.05).

The early osteogenesis effect of MC3T3-E1 cells on the diverse material surfaces was detected by using ALP activity assay (Figure 2A). At 7 d, N-Ti’s ALP activity value particularly ascended compared with that of other groups (*p* < 0.05). After 4, 7 and 14 d of osteogenic differentiation, N-Ti + L group had a lower (*p* < 0.05) ALP activity by 10%, 35%, and 56% than that on N-Ti group, respectively (Figure 2A). Meanwhile, the MC3T3-E1 cells cultured on Ti + L surface expressed less ALP by 25%, 24%, and 50% than those cultured on the Ti surface at 4, 7 and 14 d (*p* < 0.05).

ARS staining and relevant quantitative analysis were applied to attest to the formation of calcium nodes and extracellular matrix (ECM) mineralization generated by MC3T3-E1 cells on the material surfaces after 7, 14 and 21 d of osteogenic differentiation. The red calcium nodes started to emerge in the early stage of osteogenic induction at 7 d. It was found that the apparent dark red staining of minerals compounded and accumulated on N-Ti disks at 21 d (Figure 2C). By 21 d, the quantitative analysis of the ARS staining confirmed that the absorbances at 562 nm in N-Ti group reached the peak. At 7, 14 and 21 d post-culture, the absorbances were markedly enhanced by 160%, 130%, and 150% in N-Ti, and by 50%, 140%, and 130% in Ti group than those in N-Ti + L and Ti + L groups (*p* < 0.05) (Figure 2B).

**FIGURE 3:**
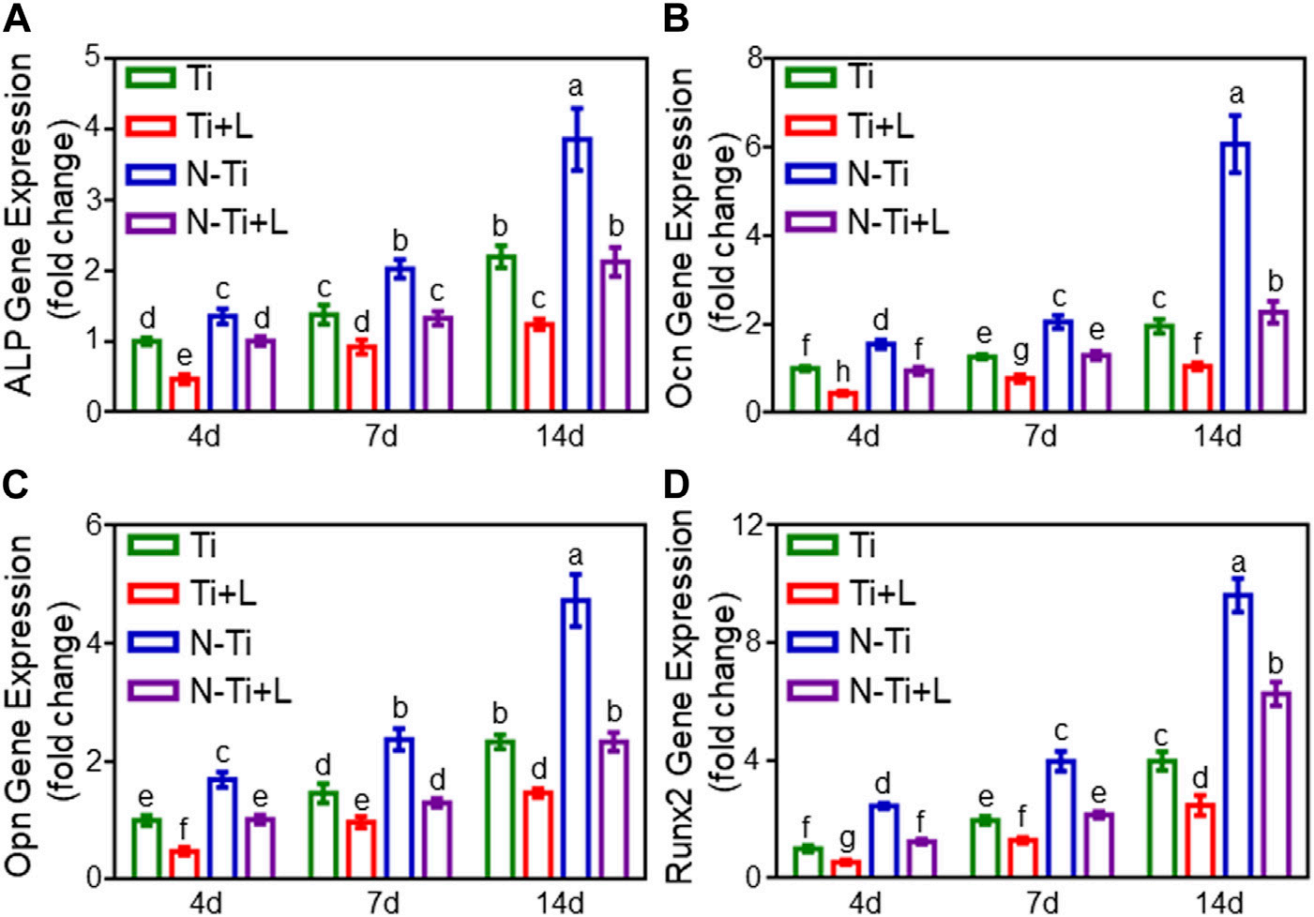
The expression of osteogenic-related genes of MC3T3-E1 cells on Ti, Ti + L, N-Ti, N-Ti + L surfaces for 4, 7, and 14 d: **(A)** ALP, **(B)** Ocn, **(C)** Opn, **(D)** Runx2. Results are shown as mean ± SD; *n* = 6/group. Values with diverse letters are notably different (*p* < 0.05).

The relative expression levels of osteogenesis-related genes were identified by using qRT-PCR (Figures 3A–D). N-Ti had a notably superior osteogenesis-associated genes expression levels in contrast to other surfaces at 14 d (*p* < 0.05). After 4 d of osteogenic differentiation, Runx2 expression resulted in a decrease of 52% in N-Ti + L, and 45% in Ti + L than that in N-Ti, and Ti, respectively (*p* < 0.05). At 14 d, ALP descended by 45% in N-Ti + L, and by 40% in Ti + L group; Ocn achieved an attenuation of 60% in N-Ti + L, and 45% in Ti + L; Opn was catabatic by 50% in N-Ti + L, and by 35% in Ti + L compared with that in N-Ti, and Ti, respectively (*p* < 0.05).

**FIGURE 4:**
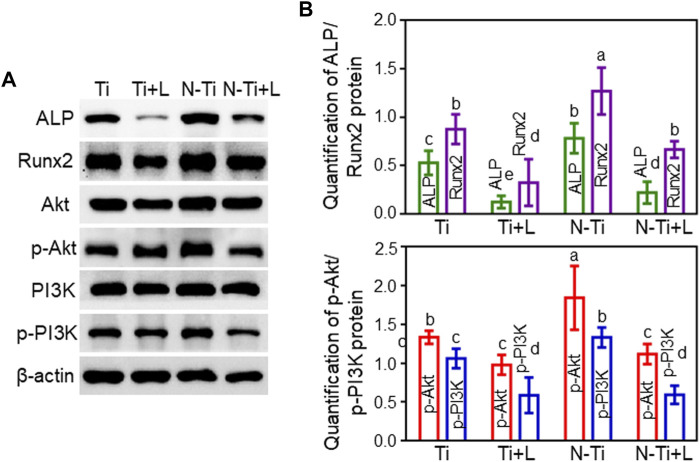
The protein expressions involved in osteogenesis and PI3K/Akt signaling pathway on Ti, Ti + L, N-Ti, N-Ti + L surfaces. **(A)** Western blot of ALP, Runx2, PI3K, p-PI3K, Akt, p-Akt, and β-actin protein expression of MC3T3-E1 cells after cultured on Ti surfaces for 7d. **(B)** Quantitative results of Western blot. Results are shown as mean ± SD; *n* = 4/group. Values with diverse letters are notably different (*p* < 0.05).

Western blot assay further assessed the osteogenesis-related proteins expression. The expression of ALP and Runx2 proteins remained at high levels at 7 d in N-Ti disks (*p* < 0.05). LY294002 leaded to a restraint of ALP and Runx2 proteins expression in N-Ti + L and Ti + L (Figures 4A,B).

### PI3K/Akt signaling pathway involvement in the osteogenic differentiation of MC3T3-E1 cells and *in vivo* osseointegration

Western blot analysis revealed the connection between the PI3K/Akt signaling pathway and the ameliorated osteogenic differentiation of MC3T3-E1 cells treated by NTAP modification. The protein bands and the quantitative analysis results of the grayscale values were rendered in Figures 4A,B. The MC3T3-E1 cells seeded on N-Ti + L and Ti + L surfaces expressed slightly diminished PI3K and Akt protein expression, and noticeably inferior p-PI3K and p-Akt protein expression compared with those on N-Ti and Ti surfaces, as confirmed by the quantitative analysis of grayscale values (*p* < 0.05).

**FIGURE 5:**
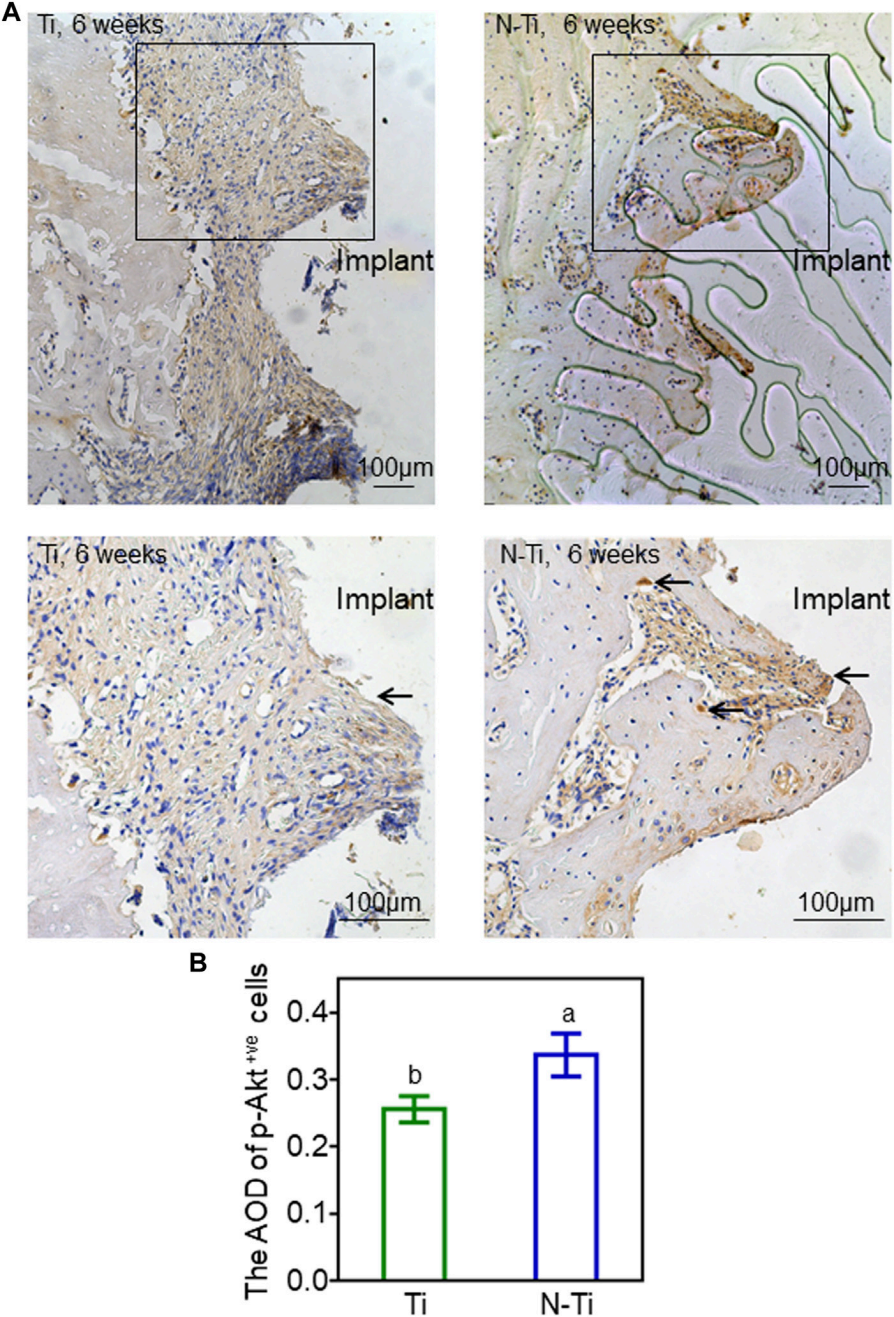
Immunochemical staining and analysis of M1 implant regions in rats. **(A)** The p-Akt staining of Ti and N-Ti samples at 6 weeks post-operation. The p-Akt-positive cells (black arrows) were located in peri-implant bone tissue. **(B)** Quantitative analysis of p-Akt expression. Results are shown as mean ± SD; *n* = 6/group. Values with diverse letters are notably different (*p* < 0.05).

The immunohistochemical staining was carried out to evaluate the expression of p-Akt around M1 implant regions. The quantitative analysis of average optical density (AOD) was performed by ImageJ software. When compared to the N-Ti group, weaker staining and fewer p-Akt-positive cells were observed in Ti group (Figure 5A). The expression of p-Akt in N-Ti was higher by 24% than that in Ti group (*p* < 0.05) (Figure 5B).

## Discussion

Recently, biomaterials, biological or synthetic substances, are introduced into body tissue and construct implanted medical devices or replace biological structures and functions. Decades of interdisciplinary research in the field of dental biomaterials resulted in the clinical success of titanium and titanium alloys implants for bone applications, as a result of their predominant biocompatible, chemical inertness, and favorable physical, mechanical properties (Agarwal and García, 2015). Even so, Ti still is a biologically inert material with a dense oxide film, inducing inadequate peri-implant’ s biological integration, and slow osteogenetic speed (Ferraris et al., 2019). The osseointegration between bone tissue and implant is the success key of implant. The process of osseointegration is complicated and time-consuming. Medical conditions (diabetes, osteoporosis, after radiotherapy, and so on) hamper bone healing and promote implant failure. Thus, surface modification is generally needed to improve Ti implants’ surfaces properties. The presence of hydrophilic Ti implant, to the great extent, can expedite peri-implant osseointegration, especially in the impaired bone condition. Novel apparatus and methods of NTAP treatment had been manufactured for Ti hydrophilic activation in the prior study (Zheng et al., 2020). It has been certified that after NTAP treatment, the water contact angle declined and the XPS analysis revealed a decrease in carbon content and an increase in oxygen content by generating functional groups such as hydroxyl groups, suggesting a superhydrophilic surface. HE staining, TRAP staining, and immunohistochemical evaluation of Ocn and Runx2 have been conducted to describe the effect of NTAP on peri-implant osseointegration. Among the prior study, we had verified that NTAP boost surface hydrophilicity, and the osteogenic activities *in vitro* and *in vivo*. The present study focused on further lucubrating the precise mechanism of NTAP’s enhancement on osteoblasts’ biological functions. In the PI3K/Akt signal pathway, PI3K could be activated by numerous genes and contribute to Akt binding to the cell membrane, and further mediates enzymatic biological effects, such as cell proliferation, apoptosis inhibition, and cell migration (Xie et al., 2019). It has been reported that PI3K/Akt pathway was involved in the differentiation and calcification of osteoblast precursor cells into mature osteoblasts (Chen J et al., 2013). Biomaterial-increased mesenchymal stem cells’ functionalities have been involved in the activation of the PI3K/Akt pathway (Chen et al., 2013). Hence, the PI3K/Akt signaling pathway and downstream targets are crucial to bone regeneration and bone remodeling. Based on this, the present study denoted the first report on the role of PI3K/Akt signaling pathway involved in the proliferation and osteogenic differentiation of MC3T3-E1 cells, and *in vivo* osseointegration on NTAP modification.

LY294002 is a generally used pharmacologic inhibitor, which works on the ATP-binding site of the PI3K enzyme, thus selectively restraining the PI3K-AKT nexus. This inhibitor has been widely applied in suppressing PI3K signaling. The reported concentration of LY294002 contained 10, 20 and 50 μM (Tsai et al., 2010; Xia et al., 2014; Li et al., 2015). The high dose of LY294002 with certain toxicity may affect cell viability and inhibit proliferation. Therefore, we chose 10 μM as the minimum effective concentration of LY294002. In the present study, the adhesion and proliferation of MC3T3-E1 cells yielded a reduction associated with PI3K inhibitor. With the extension of incubation time, the proliferative MC3T3-E1 cells substantially reduced with LY294002. These results consistently implied that PI3K inhibitor mitigated the adhesion, and proliferation of the osteoblasts on NTAP, further confirming the involvement of PI3K/Akt pathway in osteoblasts proliferation triggered by NTAP modification.

Hydrophilic surface had the ability to profit osteoblastic cells function by advancing the time of osteogenic differentiation (Zhao et al., 2005). Accordingly, this surface obtained superior osteogenic properties when compared to the conventional surface both *in vitro* and *in vivo* (Aita et al., 2009). In the early process of osteoblast adhesion, hydroxyapatite scaffolds triggered Akt to induce osteoblastic differentiation (Gemini-Piperni et al., 2014). Through activating PI3K/Akt signaling pathway, silk fibroin-based hydroxyapatite hybrid promoted the osteogenesis and osseointegration in diabetes mellitus (Ma et al., 2022). In the present study, ALP activity level, the extent of early cellular osteogenic differentiation, and the accumulation of new mineralized ECM production, strikingly descended in N-Ti + L and Ti + L disks. The highest level of ALP activity was manifested in NTAP surface, which was superior in eliciting cell responses conducive to bone formation. The matrix mineralization, and calcium nodule formation were noticeably augmented in NTAP, whereas these impacts were suppressed by LY294002 inhibitor. ALP is an early molecular marker of bone formation, and Ocn is a marker of late-stage osteogenesis and is tightly bound to osteoblast differentiation and maturation, and ECM mineral deposit (Lin et al., 2013). The noncollagenous protein Opn is also an important marker in relation to ECM formation, and Runx2 is an osteoblast transcriptional activator for osteoblast differentiation (Li and Wang, 2020). Activation of the PI3K/Akt signaling pathway boosted the expression of ALP, Runx2, and Ocn, further to facilitate the differentiation, and osteogenesis of osteoblasts on Ti surfaces (Majidinia et al., 2018). Similarly, the molecular genes (ALP, Ocn, Opn and Runx2) and proteins (ALP, and Runx2) level, involved in osteogenesis, were down-regulated in MC3T3-E1 cells cultivated with LY294002, especially in NTAP surfaces, which was consistent with the results from ALP activity and ARS staining. Collectively, these data indicated that suppressions of PI3K/Akt signaling pathway attenuated the osteogenic activities of MC3T3-E1 cells on NTAP.

The activation of PI3K/AKT signaling pathway was indispensable to the proliferation and differentiation of rat osteoblasts (Ma et al., 2010). The phosphorylation of PI3K and Akt were considerably diminished in modulating MC3T3-E1 cells’ osteoblast differentiation (Chaves Neto et al., 2011). Disrupting the Pten gene in osteoblasts, which restrained the activity of PI3K, exhibited a dramatic and progressively increasing bone mineral density throughout life (Liu et al., 2007). Some interventions, such as Zn–Sr alloy (Jia et al., 2021), and hierarchical micro-nano topography (Zheng et al., 2020), exert their osteogenic function via targeting of the PI3K/Akt signaling pathway. In this study, PI3K and Akt proteins dwindled slightly, and the levels of p-PI3K and p-Akt descended observably on N-Ti + L and Ti + L surfaces. The above results suggest that the phosphorylation of PI3K and Akt, indicators of PI3K activation, showed a significant increase in NTAP disks. LY294002 blocked the PI3K/Akt signaling pathway, and further restrained NTAP-induced osteoblast differentiation, thus reversing the effects of NTAP on MC3T3-E1 cells. As LY294002 dramatically suppressed the expression level of p-PI3K and p-Akt, the osteogenic activities in osteoblasts on NTAP were restrictive. Therefore, NTAP modification could activate the PI3K/Akt signaling pathway and thus impact greatly on the biological behaviors of osteoblasts. Furthermore, PI3K/Akt pathway is inseparable from other signaling pathways. Multiple signaling pathways, such as Wnt, MAPK and MEK/ERK signaling pathways, could regulate osteogenic differentiation. There are reciprocal connections among these pathways, which might serve as mutual signal molecules. More relevant studies need us further demonstration.

Surface modification techniques for the enhancement of osteogenic responses were demonstrated to be economical and valid approaches. It has been validated in prior study that NTAP surface modification was an effective and well-documented surface modification technique to improve the Ti surface properties with higher hydrophilicity, thus stimulating the osseointegration process, narrowing the healing time and upgrading the success in implant. In the present study, we dug deeper into the potential mechanisms by which NTAP modification can promote osteogenesis on Ti implant surfaces. With the LY294002 restraint of PI3K/Akt signaling pathway activation, MC3T3-E1 cells’ adhesion, proliferation and osteogenic differentiation *in vitro* were repressive on NTAP surfaces, which accompanied by the down-regulated expression of p-PI3K and p-Akt. The up-regulated p-Akt expression in peri-implant also was observed on NTAP-modified surfaces. All evidences suggest that the PI3K/Akt signaling pathway played an important role during the enhanced osteogenic process of NTAP-Ti surface. This result was conductive to laying a foundation for the application of NTAP treatment on implant surfaces.

## Conclusion

NTAP conduced to osteogenic activities via promoting osteoblast proliferation and differentiation by means of PI3K/Akt signaling pathway. This important finding further emphasized that PI3K/Akt signaling pathway was involved in the amelioration of osteogenesis induced by NTAP modification.

## Data Availability

The original contributions presented in the study are included in the article/Supplementary Material, further inquiries can be directed to the corresponding authors.
