# Supplementary figures and images for: Involvement of PI3K/Akt signaling pathway in promoting osteogenesis on titanium implant surfaces modified with novel non-thermal atmospheric plasma
Source: Front Bioeng Biotechnol. 2022 Sep 16;10:975840. doi: 10.3389/fbioe.2022.975840 (PMC9523010; doi:10.3389/fbioe.2022.975840)

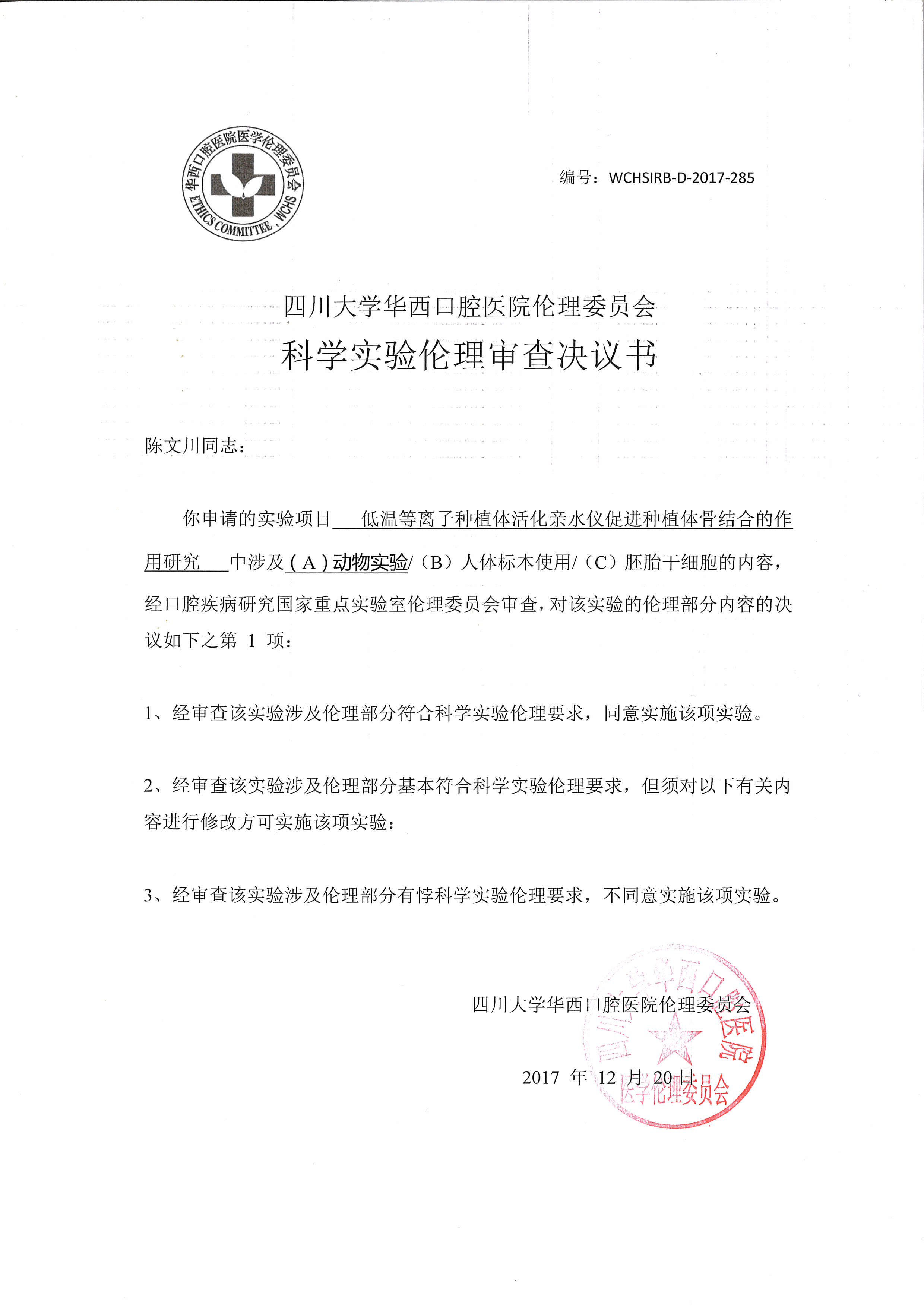

Supplement: Supplementary file 1 [file Image2.JPEG]

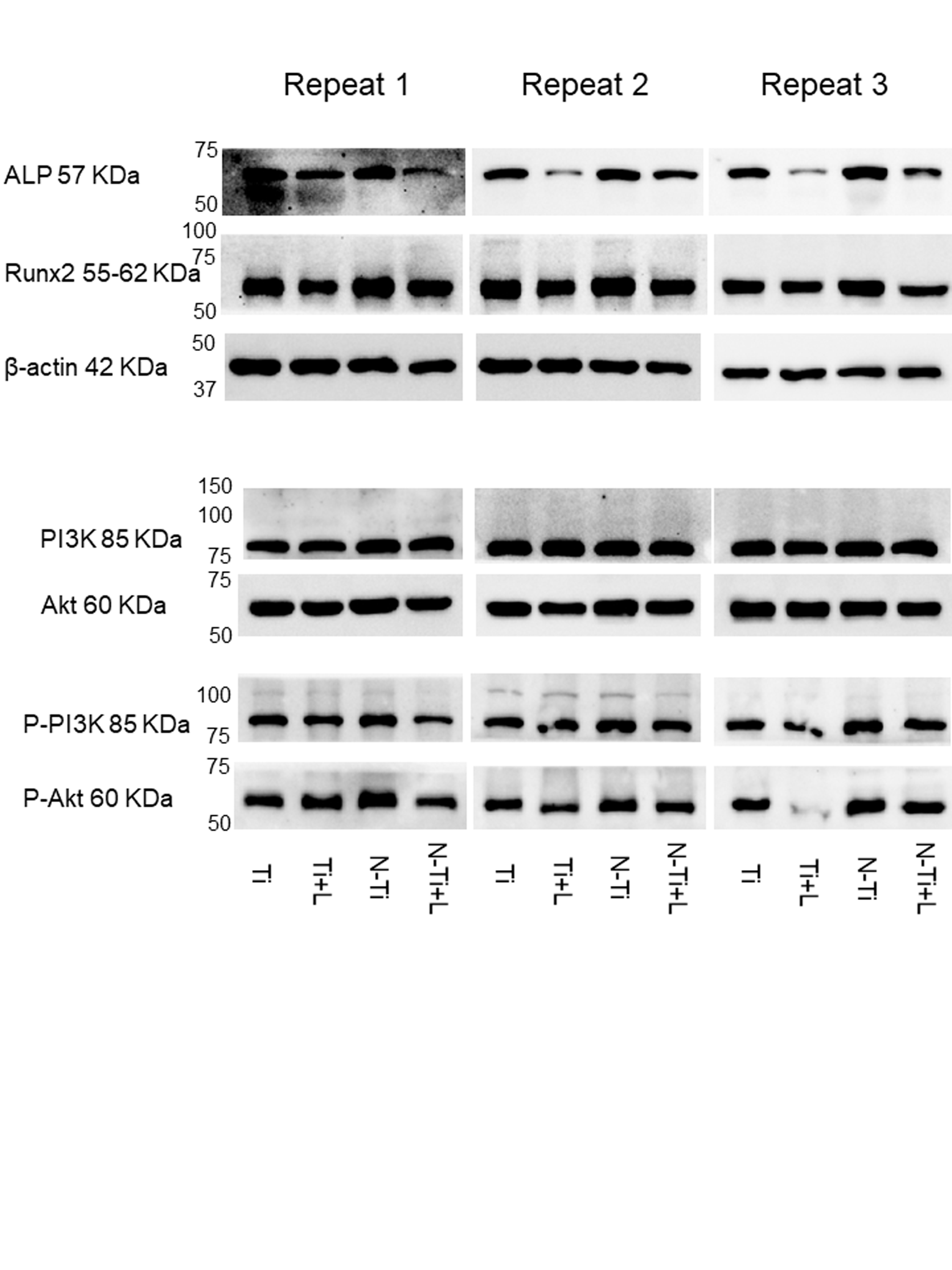

Supplement: Supplementary file 2 [file Image1.TIF]
